# Fine Needle Aspiration Biopsy of Thyroid Nodules Using Aspiration vs. Capillary Technique: Our Experience

**DOI:** 10.3390/diagnostics16132070

**Published:** 2026-07-02

**Authors:** Olatz Saenz De Argandoña Echeverría, Yensa Rodríguez, José Carlos Pariente, Francesc Vivó, Maite Rodrigo, Albert Calvo Porcel, Guillem Martín Vidal, Mattia Squarcia, Josep Puig, Núria Bargalló, Santiago Medrano-Martorell

**Affiliations:** 1 Department of Neuroradiology, Diagnostic Imaging Centre, Hospital Clínic de Barcelona, 08036 Barcelona, Spain; saenzdearg@clinic.cat (O.S.D.A.E.); albert17calvo@gmail.com (A.C.P.); guille.mavid@gmail.com (G.M.V.); squarcia@clinic.cat (M.S.); jpuig2@clinic.cat (J.P.); bargallo@clinic.cat (N.B.); medrano@clinic.cat (S.M.-M.); 2 Fundació de Recerca Clínic Barcelona (FRCB), Institut d’Investigacions Biomèdiques August Pi I Sunyer (IDIBAPS), 08036 Barcelona, Spain; pariente@recerca.clinic.cat (J.C.P.); fvivo@recerca.clinic.cat (F.V.); 3 Department of Pathology, Biomedical Diagnostic Centre (CDB), Hospital Clínic de Barcelona, Universitat de Barcelona, 08036 Barcelona, Spain; mtrodrigo@clinic.cat

**Keywords:** thyroid nodule, thyroid neoplasms, biopsy, fine-needle, ultrasonography, interventional, cytological techniques

## Abstract

**Background/Objectives**: Although ultrasound-guided fine-needle aspiration biopsy with suction (FNA-S) is preferred for evaluating thyroid nodules, the capillary technique (FNA-C) is also used. However, the diagnostic performance of both techniques remains unclear. Our objective was to compare the diagnostic performance of FNA-S versus FNA-C for thyroid nodules. **Methods**: This retrospective, observational, exploratory, single-center pilot evaluation study was conducted at a tertiary hospital from January 2023 to June 2024. A total of 157 ultrasound-guided FNA biopsies were prospectively analyzed, comprising 71 FNA-S and 86 FNA-C procedures. Clinical, ultrasound, and cytological parameters were evaluated to compare the rates of non-diagnostic samples (Bethesda I) and the necessity for repeat punctures. Bivariate analyses and multivariate logistic regression were conducted. **Results**: Both techniques exhibited comparable rates of non-diagnostic samples (28.17% for FNA-S and 27.91% for FNA-C; *p* = 0.220) and repetition rates (38.03% for FNA-S and 43.02% for FNA-C; *p* = 0.637). The only factors significantly associated with sample adequacy, regardless of techniques employed, were the location and composition of the nodule. **Conclusions**: Ultrasound-guided FNA-S and FNA-C yield similar diagnostic performance for biopsy of thyroid nodules. The choice may depend on operator preference and nodule characteristics.

## 1. Introduction

Global epidemiological studies from 2020 have shown that thyroid cancer is the most common neoplasm of the endocrine system and the ninth leading cause of cancer mortality worldwide [1]. It is the most common neoplastic disease in young adults, with a peak incidence in the sixth decade of life [2], and affects 75% of women [3]. The prevalence of thyroid cancer has increased owing to improved detection; however, mortality rates remain stable [4].

Fine-needle aspiration (FNA) is the technique of choice for cytopathological study of thyroid nodules and is recommended under ultrasound guidance. Ultrasound provides accurate and safe guidance for performing the puncture, increasing the likelihood of obtaining a sufficient sample and reducing iatrogenic complications [5,6].

The Bethesda guidelines were updated in 2023 to standardize thyroid FNA biopsies cytopathological reporting (Table 1). Bethesda category I, or “non-diagnostic,” samples lack six well-preserved and visualized groups of follicular cells, each with at least 10 cells [7]. The FNA should be repeated for Bethesda I because some studies show a 60–80% chance of a diagnostic sample [8]. The American Thyroid Association recommends no minimum interval between FNAs [9].

In addition to the ultrasound operator, the FNA technique itself includes parameters that can alter the diagnostic yield, such as lesion characteristics, needle location, sample, the needle thickness, aspiration technique, and presence of a cytotechnician in the puncture room [Rapid On-Site evaluation (ROSE)]. Although the Bethesda guidelines recommend an unsatisfactory sample rate below 10% [7], rates in routine clinical practice can reach 40% [10].

Several studies have compared the diagnostic yield and safety of FNA with needle positioning relative to the ultrasound transducer, known as the short- and long-axis needle techniques [11]. However, few comparative studies have examined aspiration and capillary suction sampling methods [12,13,14,15,16]. In line with the literature, we used the term FNA broadly to refer to any fine-needle sampling technique. Because “aspiration” can be confusing, we differentiated between FNA with suction (FNA-S), the classical syringe technique, and FNA with capillary (FNA-C). In FNA-S, the needle is advanced to several points in the target nodule and aspirated (with or without a vacuum system) up to five times before withdrawal. In FNA-C, the needle is moved to several target nodule points without suction. The needle can be rotated on its axis until a sufficient sample is obtained in the lumen. Here, cells are detached by needle puncture, reducing blood contamination [17].

Despite the potential advantages proposed for each technique, the available evidence remains inconclusive. Comparative studies have not consistently demonstrated the superiority of either FNA-S or FNA-C in terms of sample adequacy or diagnostic accuracy, and the optimal sampling approach remains a matter of debate [18].

Therefore, we designed a study to compare the diagnostic performance of ultrasound-guided FNA-S and FNA-C.

## 2. Materials and Methods

The project received approval from the ethics committee of our institution (HCB/2025/0032), which serves as a center of reference for managing thyroid pathology. We conducted a retrospective, prospectively ascertained observational study analyzing 157 FNABs. Punctures were performed between 1 January 2023 and 30 June 2024 on patients with thyroid nodules detected by palpation and referred to our center for ultrasound examination. All consecutive patients who met the inclusion criteria during the study period were included. The indication for FNA was determined according to the EU-TIRADS system, based on nodule characteristics [19]. Ultrasound examinations were performed using a Canon Aplio i800 ultrasound scanner (Canon Medical Systems Corporation, Otawara-shi, Japan), and the punctures were performed with 2-inch long, 25G hypodermic needles. Due to the retrospective nature of the study, the sampling technique (FNA-S vs. FNA-C) was not randomized but was determined by the attending radiologist based on real-time assessment of nodule characteristics (vascularity, depth, cystic component) and operator preference, reflecting real-world clinical practice.

**Figure 1:**
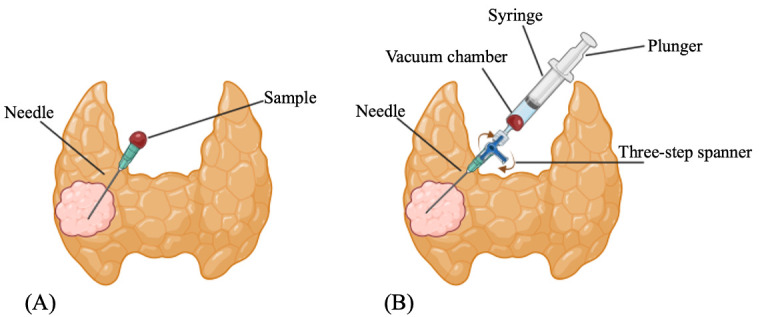
Material mounting system of ultrasound-guided fine-needle aspiration for thyroid nodule biopsy. (**A**) In capillary puncture, a 25G, 40 mm hypodermic needle is required. (**B**) In aspiration puncture, a 25G, 40 mm hypodermic needle is connected to a vacuum system consisting of a 10 cc syringe and a three-step spanner. The vacuum system can be held in place by positioning the needle cap between the plunger’s support and base.

For FNA-C, a 25G, 40 mm hypodermic needle was used without a vacuum system or syringe. The needle was inserted into the solid area and advanced to several points within the target nodule, or rotated on its axis, thereby allowing cellular material to enter the lumen spontaneously due to surface-tension forces (capillary effect). The procedure stopped once material was visible in the reservoir (hub), avoiding prolonged aspiration that could induce coagulation (Figure 1).

In punctures using FNA-S, the same needle (25G, 40 mm) was used and connected to a vacuum system with a 10 cc syringe and three-step spanner. Once positioned inside the solid portion of the nodule, the spanner was closed and then opened to generate constant negative pressure. Back-and-forth movements were made with slight changes in needle direction, allowing cellular material from different nodule areas to be collected. When the sample was visible in the needle reservoir (hub), negative pressure was released by closing the valve before removing the needle from the nodule (Figure 1).

Five radiologists with 3–15 years of experience performed the punctures. Between one and three passes were made per nodule, with the exact number not recorded due to the retrospective nature of the study and missing data in clinical reports. The decision to use the same or an alternative technique when repeating a non-diagnostic FNA was not standardized and depended on the operator’s judgment at the time of the procedure. FNA-C was preferentially used for hypervascular nodules or lesions in close proximity to major cervical vessels to minimize hemorrhagic artifact and blood contamination; for superficial nodules where capillary forces are sufficient to obtain material; and for patients with high bleeding risk. FNA-S was generally chosen for deeply located nodules or those with a firm/fibrotic consistency, where negative pressure facilitates cell detachment; predominantly solid nodules where active suction may improve cellular yield; and cystic or predominantly cystic nodules, to aspirate fluid content and obtain mural cells.

A cytotechnician occasionally assisted with sample collection. Data were collected retrospectively from medical records. The analyzed parameters were patient age and sex, affected thyroid lobe and nodule location, nodule composition and ultrasound characteristics, EU-TIRADS classification, inter-observer variability, number of passes, sample acquisition technique, and need for repeat FNA. Nodules in the posterior sectors or the lower third of the lobes, and those <10 mm, were considered “difficult punctures”.

Statistical analysis was performed using Python 3.11 (pandas 2.0.3, SciPy 1.13.1, statsmodels 0.14.0, and scikit-learn 1.5.2). The distribution of continuous variables was assessed with the Shapiro–Wilk test and visual inspection of histograms; all continuous variables are summarized as median (interquartile range) and were compared between techniques (FNA-S vs. FNA-C) using the two-tailed Mann–Whitney U test. To compare categorical parameters between techniques, we performed a two-tailed Fisher’s exact test or a Pearson chi-square test of independence across all subgroups of a given characteristic, depending on the expected cell frequencies, and pooled both techniques with confidence intervals (95% CI, *p*-value < 0.05) to quantify the relative risk of obtaining an insufficient sample with each technique. Candidate predictors were first screened by univariable logistic regression and subsequently entered into a multivariable logistic regression model, with results expressed as adjusted odds ratios and 95% confidence intervals; the discriminative performance of the model was evaluated using the area under the receiver operating characteristic (ROC) curve. All tests were two-sided, and a *p*-value < 0.05 was considered statistically significant.

## 3. Results

No differences were observed between FNA-C and FNA-S techniques for demographic or lesion-related variables. Among patients who underwent FNA, 122 were female, aged 20 to 89 years. The proportion of female patients was comparable (75.6% vs. 80.3%), as was mean age (59.7 ± 15.1 vs. 59.9 ± 13.8 years). In 71 punctures, samples were obtained using FNA-S, and in the remaining 86, samples were obtained using FNA-C. Nodule dimensions showed no significant differences between groups (Table 2). Lesion distribution by thyroid lobe (isthmus 7% vs. 5.6%; lobes 93% vs. 94.4%) and pole (inferior 47.7% vs. 38.0%; middle 27.9% vs. 29.6%; superior 24.4% vs. 32.4%) showed no significant variation. Echogenicity patterns (hyperechoic 64% vs. 56.3%; hypoechoic 31.4% vs. 39.4%) and EU-TIRADS categories (T2 3.5% vs. 5.6%; T3 51.2% vs. 40.9%; T4 31.4% vs. 40.9%; T5 14% vs. 12.7%) were similarly distributed.

Punctures per nodule and distribution among radiologists who performed the procedure showed no differences between techniques. Both methods were applied to comparable patient and lesion profiles (Table 2). Significant differences in lesion depth were observed (*p* = 0.001), with a higher percentage of samples in lateral and medial lesions. In lateral lesions, more significant samples were obtained with both FNA-C and FNA-S, whereas in more medial lesions, only FNA-S yielded significant samples (lateral, 44.19% vs. 35.21%; medial, 12.79% vs. 38.03%; posterior, 17.44% vs. 16.90%; superficial, 25.58% vs. 9.86%). Significant differences were detected in biopsied nodule composition (*p* = 0.023), with puncture being more efficient in solid nodules (65.12% vs. 52.11%) and solid-cystic nodules (33.72% vs. 35.21%). A tendency was observed between techniques in the two-pass subgroup, with higher non-diagnostic samples in the FNA-S group than in the FNA-C group (50% vs. 18.5%), though this did not reach significance (Table 3).

No parameters showed significant differences in sample viability and sufficiency between FNA-S and FNA-C. The rates of insufficient samples (Bethesda I, 28.17% for FNA-S vs. 27.91% for FNA-C; *p* = 0.220), procedure repetition (38.03% for FNA-S vs. 43.02% for FNA-C; *p* = 0.637), and “difficult punctures” (27.91% for FNA-S vs. 29.63% for FNA-C; *p* > 0.9) were similar between techniques.

## 4. Discussion

We compared FNA-S and FNA-C, two ultrasound-guided techniques used in clinical settings, in a retrospective, observational, exploratory, single-center pilot evaluation study. Although FNA has proven to be the most cost-effective first diagnostic tool for thyroid nodules, there is ongoing controversy over whether any single technique is superior in diagnostic performance [12]. In the absence of clear consensus in clinical guidelines, the decision often relies on the operator’s experience and the nodule’s characteristics. FNA-C has been recommended for its simplicity and reduced post-traumatic blood residue [13]. In FNA-C, cells are detached by mechanical needle cutting and drawn into the lumen by capillary forces, thereby minimizing reactive changes such as hemorrhage, endothelial hyperplasia, thrombosis, fibrosis, cystic transformation, or infarction [14]. Because it reduces blood contamination, FNA-C is considered most suitable for highly vascularized nodules [15].

Both techniques showed similar rates of non-diagnostic samples, with an overall percentage of approximately 28%. These findings align with previous studies reporting inadequacy rates of around 5–20% for both techniques. In the two-pass subgroup, a trend was observed between techniques: the FNA-S group had a higher rate of non-diagnostic samples than the FNA-C group, although this difference was not statistically significant. The analysis of pass numbers revealed a significant association with sample adequacy. However, the absence of significance in the subgroup analysis is likely due to limited statistical power, suggesting that a larger sample size might have demonstrated a significant difference. In secondary analysis, Shin et al. identified notable differences in nodules considered categorized as “difficult to puncture”, defined as those smaller than 10 mm and located within 3 mm of critical structures. These results showed that aspiration produced more favorable outcomes, including fewer non-diagnostic samples and no complications [16]. In line with Shin et al. and in contrast to our findings, another study by Hatami et al. reported a higher proportion of diagnostically insufficient cell clusters and higher rates of nondiagnostic and inadequate specimens in the FNC group than in the FNA group [20].

The diagnostic adequacy of thyroid nodule characteristics is a topic of considerable debate. Significant differences in sample viability were observed based on nodule location and composition. Nodules located laterally or medially, as well as those with solid or solid-cystic compositions, yielded higher amounts of adequate cytological material, regardless of the technique used. These findings are consistent with those of Inci et al., who reported a higher proportion of unsatisfactory samples in heterogeneous nodules characterized by degeneration, cystic areas, or intralesional necrosis, particularly in elderly patients [21]. In contrast to our study, they did not find a significant correlation between nodule size and sample adequacy. Reduced diagnostic performance for cytological procedures has been associated with nodules larger than 10 mm, those exhibiting hypoechogenicity, a lack of vascularization, and high stiffness on elastography [22].

The influence of the number of passes on cytological adequacy was not assessed because of the study’s retrospective design; however, several studies have underscored its importance. The National Cancer Institute recommends conducting two to five passes per nodule to ensure sufficient sampling, reduce hemorrhagic artifacts, and enhance cell yield. This recommendation is based on studies demonstrating adequacy rates exceeding 85% without increased procedural morbidity [23].

The number of passes may depend on rapid on-site evaluation (ROSE), a method proposed to improve the adequacy of cytological samples during FNA, particularly in centers with high baseline rates of unsatisfactory samples. Studies indicate that ROSE can decrease the rate of inadequate samples by 12% to 30.5% and improve material quality [18]. This method is particularly advantageous in settings with technical or experience limitations, as it may enhance the safety and efficacy of FNA procedures. Although a single universal number of passes cannot be established, evidence suggests that 2 to 5 passes are safe and effective in routine practice, especially when immediate evaluation is unavailable [24].

Fine-gauge needles (25G), as used in this study, are associated with lower bleeding rates and improved morphological quality. When aspirated material is hemorrhagic, complete inclusion in a cell block significantly enhances diagnostic performance, particularly in procedures without a cytotechnician. This technique increases sensitivity, minimizes the need for repeat punctures, and is cost-effective in settings with a high incidence of non-diagnostic samples [25].

Our study has limitations that merit comment. The small sample size limited the generalizability and statistical power of our findings. We did not exclude punctures of cystic/spongiform nodules (EU-TIRADS 2). The number of needle passes per sample, which correlates with profitability, was reported inconsistently and was therefore excluded from the analysis. The decision about the technique to use depended on the operator’s judgment at the time of the procedure. Additionally, our non-diagnostic rate of approximately 28% is higher than the 5–20% range recommended by the 2023 Bethesda system [10]. This likely reflects our real-world clinical setting, which lacks routine ROSE, includes all consecutive patients without excluding difficult punctures, and involves operators with variable experience (3 to 15 years). Comparable inadequacy rates have been reported in other real-world series without ROSE [11,21].

In conclusion, our study showed that both techniques yielded similar non-diagnostic rates (FNA-S: 28.2%, FNA-C: 27.9%; *p* = 0.220) and repeat puncture rates (38.0% vs. 43.0%; *p* = 0.637). Sample adequacy was significantly associated with nodule location (*p* = 0.001) and composition (*p* = 0.023), but not with the technique used. Insufficient sampling was associated with nodule locations that were either lateral or medial, as well as with lesions containing a higher proportion of solid components. The choice between the two methods may depend on the operator’s familiarity and the nodule’s specific characteristics. To confirm these findings, future studies involving larger cohorts are necessary.

## Figures and Tables

**Table 1:** Bethesda System for Reporting Thyroid Cytopathology (adapted from reference [10]).

Diagnostic Category	Risk of Malignancy (%)	Usual Management
I (Not diagnostic)	5–20	Repeat US-guided FNA
II (Benign)	2–7	Clinical and ultrasound follow-up
III (Atypical with uncertain significance)	13	Repeat FNA, molecular study, diagnostic lobectomy or active surveillance
IV (Follicular neoplasm)	23	Molecular study, diagnostic lobectomy
V (Suspicious for malignancy)	67–83	Molecular study, lobectomy or subtotal thyroidectomy
VI (Malignant)	97–100	Lobectomy or subtotal thyroidectomy

**Table 2:** Univariate analysis between ultrasound-guided fine-needle aspiration techniques for thyroid nodules. FNA-C: capillary technique. FNA-S: suction technique. SD: standard deviation. Continuous variables are reported as mean (SD) and compared using Welch’s two-sample *t*-test (unequal variances not assumed). Binary variables are reported as *n* (%) and compared using the two-tailed Fisher’s exact test. Categorical variables with ≥3 categories are reported as *n* (%) and compared using Pearson’s chi-square test of independence. *p*-values with an asterisk indicate *p* < 0.05.

Variable	Whole Cohort (*n* = 157)	FNA-C (*n* = 86)	FNA-S (*n* = 71)	*p*-Value
Age, mean (SD) [years]	59.82 (14.49)	59.92 (15.14)	59.69 (13.76)	0.919
Volume, mean (SD) [mm^3^] †	7279.66 (13,446.97)	6556.10 (8283.14)	8164.01 (17,921.75)	0.581
Largest transversal diameter, mean (SD) [mm]	20.64 (10.15)	21.46 (9.80)	19.64 (10.54)	0.268
Largest anteroposterior diameter, mean (SD) [mm]	17.19 (8.38)	17.26 (8.47)	17.09 (8.32)	0.899
Largest longitudinal diameter, mean (SD) [mm] †	24.58 (11.87)	24.19 (11.86)	25.05 (12.01)	0.718
Gender, female, *n* (%)	122 (77.71)	65 (75.58)	57 (80.28)	0.565
Repeated puncture, *n* (%)	64 (40.76)	37 (43.02)	27 (38.03)	0.625
Side location, *n* (%)				0.637
Right	84 (53.50)	44 (51.16)	40 (56.34)	
Left	61 (38.85)	34 (39.53)	27 (38.03)	
Isthmus	12 (7.64)	8 (9.30)	4 (5.63)	
Lobulation/Pole location, *n* (%)				0.497
Isthmus	9 (5.73)	5 (5.81)	4 (5.63)	
Lobe	148 (94.27)	81 (94.19)	67 (94.37)	
Inferior	68 (45.95)	41 (50.62)	27 (40.30)	
Middle	38 (25.68)	21 (25.93)	17 (25.37)	
Superior	42 (28.38)	19 (23.46)	23 (34.33)	
Location, *n* (%)				0.001 *
Lateral	63 (40.13)	38 (44.19)	25 (35.21)	
Medial	38 (24.20)	11 (12.79)	27 (38.03)	
Posterior	27 (17.20)	15 (17.44)	12 (16.90)	
Superficial	29 (18.47)	22 (25.58)	7 (9.86)	
Radiologist entrusted with the procedure, *n* (%)				0.108
#1	60 (38.22)	33 (38.37)	27 (38.03)	
#2	8 (5.10)	5 (5.81)	3 (4.23)	
#3	19 (12.10)	8 (9.30)	11 (15.49)	
#4	28 (17.83)	11 (12.79)	17 (23.94)	
#5	42 (26.75)	29 (33.72)	13 (18.31)	
EU-TIRADS classification, *n* (%)				0.510
2	7 (4.46)	3 (3.49)	4 (5.63)	
3	73 (46.50)	44 (51.16)	29 (40.85)	
4	56 (35.67)	27 (31.40)	29 (40.85)	
5	21 (13.38)	12 (13.95)	9 (12.68)	
Nodule composition, *n* (%)				0.023 *
Spongiform	4 (2.55)	0 (0.00)	4 (5.63)	
Cystic	6 (3.82)	1 (1.16)	5 (7.04)	
Solid	93 (59.24)	56 (65.12)	37 (52.11)	
Solid-cystic	54 (34.39)	29 (33.72)	25 (35.21)	

† Volume and largest longitudinal diameter (CC) were available for *n* = 100 of 157 subjects; means and *t*-tests are computed over available observations only. Pole percentages (Inferior, Middle, Superior) are computed over lobar nodules only (*n* = 148).

**Table 3:** Sample Insufficiency Rates by Nodule Characteristic and Aspiration Technique. Insufficiency is defined as a Bethesda category I (non-diagnostic) result. *p* (technique): two-tailed Fisher’s exact test comparing the two techniques within each subgroup. *p* (subgroups): Pearson χ^2^ test across all subgroups of a given characteristic, pooling both techniques. Values with an asterisk indicate *p* < 0.05.

Characteristic	Subgroup	FNA-S Insufficient/Total (%)	FNA-C Insufficient/Total (%)	*p* (Technique)	*p* (Subgroups)
Overall	All samples (*n* = 157)	20/71 (28.2%)	24/86 (27.9%)	1.000	—
Localization					0.203
	Lateral	8/25 (32.0%)	14/38 (36.8%)	0.790	
	Medial	9/27 (33.3%)	3/11 (27.3%)	1.000	
	Posterior	3/12 (25.0%)	2/15 (13.3%)	0.628	
	Superficial	0/7 (0.0%)	5/22 (22.7%)	0.296	
Echogenicity					0.509
	Hyperechoic/Isoechoic	11/40 (27.5%)	13/55 (23.6%)	0.811	
	Hypoechoic	7/28 (25.0%)	10/27 (37.0%)	0.391	
	Markedly Hypoechoic	2/3 (66.7%)	1/4 (25.0%)	0.486	
Pole					0.461
	Inferior	7/27 (25.9%)	11/41 (26.8%)	1.000	
	Isthmus	2/4 (50.0%)	1/5 (20.0%)	0.524	
	Middle	6/17 (35.3%)	8/21 (38.1%)	1.000	
	Superior	5/23 (21.7%)	4/19 (21.1%)	1.000	
No. of Passes					0.013 *
	1 pass	11/51 (21.6%)	12/37 (32.4%)	0.327	
	2 passes	6/12 (50.0%)	5/27 (18.5%)	0.061	
	3 passes	2/7 (28.6%)	4/19 (21.1%)	1.000	
	4 passes	1/1 (100.0%)	3/3 (100.0%)	—	

## Data Availability

The original contributions presented in this study are included in the article. Further inquiries can be directed to the corresponding author.
